# Understanding the Use of Mobility Data in Disasters: Exploratory Qualitative Study of COVID-19 User Feedback

**DOI:** 10.2196/52257

**Published:** 2024-08-01

**Authors:** Jennifer Lisa Chan, Sarah Tsay, Sraavya Sambara, Sarah B Welch

**Affiliations:** 1 Department of Emergency Medicine Feinberg School of Medicine Northwestern University Chicago, IL United States; 2 Department of Emergency Management University of San Diego Health San Diego, CA United States; 3 Harvard University Boston, MA United States; 4 Buehler Center for Health Policy and Economics Feinberg School of Medicine Northwestern University Chicago, IL United States

**Keywords:** mobility data, disasters, surveillance, COVID-19, qualitative, user feedback, policy making, emergency, pandemic, disaster response, data usage, situational awareness, data translation, big data

## Abstract

**Background:**

Human mobility data have been used as a potential novel data source to guide policies and response planning during the COVID-19 global pandemic. The COVID-19 Mobility Data Network (CMDN) facilitated the use of human mobility data around the world. Both researchers and policy makers assumed that mobility data would provide insights to help policy makers and response planners. However, evidence that human mobility data were operationally useful and provided added value for public health response planners remains largely unknown.

**Objective:**

This exploratory study focuses on advancing the understanding of the use of human mobility data during the early phase of the COVID-19 pandemic. The study explored how researchers and practitioners around the world used these data in response planning and policy making, focusing on processing data and human factors enabling or hindering use of the data.

**Methods:**

Our project was based on phenomenology and used an inductive approach to thematic analysis. Transcripts were open-coded to create the codebook that was then applied by 2 team members who blind-coded all transcripts. Consensus coding was used for coding discrepancies.

**Results:**

Interviews were conducted with 45 individuals during the early period of the COVID-19 pandemic. Although some teams used mobility data for response planning, few were able to describe their uses in policy making, and there were no standardized ways that teams used mobility data. Mobility data played a larger role in providing situational awareness for government partners, helping to understand where people were moving in relation to the spread of COVID-19 variants and reactions to stay-at-home orders. Interviewees who felt they were more successful using mobility data often cited an individual who was able to answer general questions about mobility data; provide interactive feedback on results; and enable a 2-way communication exchange about data, meaning, value, and potential use.

**Conclusions:**

Human mobility data were used as a novel data source in the COVID-19 pandemic by a network of academic researchers and practitioners using privacy-preserving and anonymized mobility data. This study reflects the processes in analyzing and communicating human mobility data, as well as how these data were used in response planning and how the data were intended for use in policy making. The study reveals several valuable use cases. Ultimately, the role of a data translator was crucial in understanding the complexities of this novel data source. With this role, teams were able to adapt workflows, visualizations, and reports to align with end users and decision makers while communicating this information meaningfully to address the goals of responders and policy makers.

## Introduction

### Background

The COVID-19 pandemic has had a significant impact on the world, with more than 6.82 million deaths and more than 670 million cases globally as of February 2023 [1]. Public health measures, specifically physical distancing policies, were implemented throughout the world in 2020, playing a major role in the early phases of the COVID-19 global pandemic [2-4]. In general, these policies were based on the assumption that individual and population movement dynamics of those infected with COVID-19 were likely to impact the degree of spread. Studies have drawn a strong association between public health measures (eg, stay-at-home orders, physical distancing requirements) and reductions in population movement. Implementing distancing policies during the prevaccine era had the potential to mitigate the spread of disease and ultimately decrease demand on health care systems [5,6].

Nations took varying approaches to enacting COVID-19 public health measures. The People’s Republic of China implemented travel restrictions and widespread population-based quarantine measures until the end of 2022. Italy, affected by COVID-19 in February 2020, instituted a nationwide quarantine until mid-2021 [7]. The United States took a patchwork approach to social distancing policies, which varied across the country by jurisdiction. Some cities had strict social distancing policies while others did not institute a stay-at-home order. During the latter part of 2020 and into 2021, the lack of a coordinated nationwide approach left cities and states to independently determine when to relax or reinstitute policies during subsequent epidemiologic waves due to new COVID-19 variants.

Using data in disasters involves a wide array of processes and skills in order to result in effective use and impact on decision-making. The process of using data includes data collection, processing, analyses, product creation (eg, visualization), and sharing [8,9]. An emerging body of evidence now recognizes the importance of collaboration, communication, and data literacy, all human factors, as important to effective use of data [10]. For government agencies around the world, understanding near real-time movement of individuals and groups was extremely difficult [11]. Key decision makers had to establish timely policies with limited, evolving information [12]. Physical distancing policies faced the same, if not more nuanced, challenges in using timely evidence, as the data and information that represented population-based movements were emerging from novel data sources not previously used for a pandemic of unprecedented proportions. Human mobility data, which are passively generated by digital devices that track information over both time and geography, provide a unique data source that reflects human movement in disaster settings [13]. In 2010, mobility data predicted population movement after the Haiti earthquake, and early studies in 2020 showed that stay-at-home orders were associated with decreased population movement [14].

In response to the opportunities that mobility data presented for decision-making in the early phase of the COVID-19 pandemic, these data were analyzed by researchers from a wide range of disciplines with the intention of understanding disease dynamics as well as the impact of physical distancing policies on human movement at the national, regional, and local levels [15]. Klein et al [16] characterized physical distancing behaviors in the United States during the autumn of 2020, highlighting urban-rural differences and concluding that physical distancing policies were associated with changes in movement patterns. Much of the current research has focused on analyzing such data and anticipating the influence on policy making, including how the applications with these quantitative data were able to predict disease spread or identify future outbreak locations [17,18]. Although movement data had been used by governments in the past, use during the COVID-19 pandemic posed new challenges [19].

A network of global researchers, response planners, and policy makers collaborated in early 2020 with the aim of translating mobility data in order to positively impact response planning and policy making. With access to anonymized and privacy-preserving mobility data from Facebook (Meta), the COVID-19 Mobility Data Network (CMDN) facilitated the responsible and meaningful use of widely available human mobility data by policy makers around the world [20,21]. Mobility data sets were shared with trusted partners (eg, researchers) to collaborate with the public sector to inform response and recovery. Facebook also created a differential privacy framework to protect the privacy of individuals in aggregated data sets, informing public sector response to the COVID-19 pandemic. Over 150 researchers from around the world joined the network, contributing their scientific and analytic skills to develop methods necessary to transform these new data sources to meet the needs of their government counterparts. Both researchers and policy makers assumed that collecting, analyzing, and sharing mobility data would provide insights to help both policy makers and response planners guide their activities to mitigate the human, economic, and social impacts of COVID-19.

Despite the ability to collect, analyze, and share mobility data results around the world, there lacked evidence for how mobility data were used in response planning and policy making. There also lacked evidence and knowledge of which processes or steps in this applied research were more or less effective. Recent evaluations of mobility studies in COVID-19 have shown that applied research, or translational research, is often neglected, limiting the ability of decision makers and the public to take action and benefit from these efforts. In the outbreak modeling community, Nixon et al [19] reviewed 136 papers but found that only 1 in 4 papers evaluated the performance of the models and few disclosed the uncertainty of the data and transparency of the methodology. In addition, more than 50% of the studies focused on predictions at the national level when policy makers and planners more frequently focus on local-level decision-making, and these approaches were least useful for decision-making [19]. Although quantitative methods can, to some degree, provide evidence on the accuracy of mobility data, complementary qualitative methods can provide evidence of their use in the social dynamic environments of disasters.

### Study Objectives

This study focused on advancing the understanding of how researchers, policy makers, and response planners used human mobility data during the COVID-19 pandemic for the purpose of supporting decision-making related to stay-at-home orders and social distancing policies. Using qualitative methods to investigate the near real-time and lived experiences and perceptions of those using mobility data, the study used a constructivist point of view to describe experiences of researchers and practitioners who used these data in response planning and policy making. The study aimed to identify themes related to what would make the data more usable and valuable for public health practitioners, response planners, and researchers in disaster and emergency settings.

This study was framed within the information management and disaster response fields. The role of data and information management in disaster contexts is tightly linked to situational awareness. As described by Vieweg [22] and others [23,24], situational awareness refers to understanding the disaster environment, including hazards and emerging threats; they also described that improved situational awareness enables informed decisions related to response and recovery activities. In addition, data, whether traditional or novel, are perceived as valuable and useful when results can improve an individual’s or group’s situational awareness of the disaster environment. Situational awareness is assumed to be dynamic and often constantly changing, and uncertainty of the disaster environment is common.

Specific aims of the study were to explore the following:

- How groups use human mobility data and the results in response planning and policy making
- What processes and ways of working were used to analyze human mobility data and visualize or communicate the results
- Human factors related to processes described in the previous bullets, including various purposes for using mobility data, communication dynamics, and translational activities that enable the use of the data

## Methods

### Ethical Considerations

This project used preexisting interview information acquired for Crisis Ready’s User Feedback Project and collected for the purposes of project assessment and evaluation. As such, the initial data collection was not reviewed by an institutional review board and did not collect informed consent from interview participants. This exploratory study used these data to understand how mobility data in the CMDN were being used during a large-scale global public health disaster. It was reviewed by the Northwestern University institutional review board and deemed not human research, as it was secondary data analysis with no identifiers (STU00214214).

### Approach

Our study was grounded in a phenomenology approach to understand how individuals were making sense of novel mobility data and incorporating the data into preexisting workflows. The unprecedented and highly uncertain and dynamic environment experienced by the interviewees both due to the COVID-19 pandemic and novel data source was best aligned with this methodological approach. Our analytical approach used thematic analysis to identify patterns of behavior and opinion as well as understand how individuals navigated the many new inputs during this time. The analysis team consisted of 3 individuals (JLC, ST, SBW). JLC is an emergency medicine physician, humanitarian practitioner, data specialist, and researcher with qualitative experience. ST is an emergency management professional with over a decade of experience at the intersection of public health, health care, and emergency management. SBW is a public health evaluator and research project manager with experience in qualitative methods.

### Data Collection

Study data included interviews completed with individuals participating in the CMDN and selected using a combination of convenience and purposive sampling to have global representation and to enroll participants from different sectors (ie, public health practitioners, response planners, or researchers) to include a variety of experiences and perspectives. Interviews were deidentified and professionally transcribed before being shared with the study team, who uploaded the data to Dedoose Version 9.0.17. Study data were defined as narrative data from interviews and demographic information.

### Analysis

We used thematic analysis with an inductive approach to understand our data. ST and SBW independently open-coded 2 transcripts each, assigning codes to all concepts identified in the interview data. The entire team (JLC, ST, SBW) met to review the initial codebook and refine it by combining similar concepts from approaches derived from the literature [25-27]. ST and SBW then exchanged transcripts and blind double-coded them using the initial codebook. The entire team refined the codebook further and blind triple-coded a fifth transcript. The codebook was refined and finalized (Multimedia Appendix 1).

ST and SBW blind double-coded all interview transcripts in a systematic manner guided by coding practices outlined by MacQueen et al [26]. Coding discrepancies were documented and discussed, and the code was changed to reflect the agreed-upon code and placement. If ST and SBW could not come to an agreement, JLC was engaged in the conversation, and discussion continued until consensus between the entire coding team was reached using previously published codebook methods for team approaches to coding [28,29]. All coded excerpts were reviewed by code. Team members reviewed each quote excerpt and summarized and organized them into themes and concepts to look for common experiences, examples, and opinions. 

To increase the validity, initial project findings were presented to stakeholders of participants in CMDN and peer researchers before the conclusion of analysis. This activity included peer review activities and a hybrid member-check and data party [30-32]. The analysis team conducted 2 meetings over the course of 2 days where project overview, transcript excerpts, and findings were presented to the group. Participants were asked if the information resonated with them, made sense, and reflected their understanding based on their experience; they were also asked to share their thoughts for next steps for using these data for application in future emergencies. Feedback from this meeting was used to check the themes developed in the final analysis. There were no additional themes identified based on the feedback.

## Results

### Participants

The anonymized and deidentified data set consisted of 33 interviews conducted with 45 researchers and practitioners involved in CMDN from June 2020 to September 2020. The majority of interviewees were from North America (28/45, 62%), with a predominance from the United States (26/45, 58%), followed by Asia (11/45, 24%; Table 1). Of the interviewees, 62% (28/45) were men, and 38% (17/45) were women. In addition, 47% (21/45) of the interviewees had practitioner roles, while 42% (19/45) had primary research roles. Specific interviewees’ roles included state-level Chief Information Officer, consultant supporting a state-level response, data scientist in city government, emergency manager, and assistant professor of epidemiology. A few interviewees described their roles as hybrid, encompassing both researcher and practitioner activities.

**Table 1:** Participant demographics (n=45).

Characteristics			Results, n (%)
**Gender**			
	Male	28 (62)	
	Female	17 (38)	
**Continent**			
	Africa	1 (2)	
	Asia	11 (24)	
	Europe	2 (4)	
	North America	28 (62)	
	South America	3 (7)	
**Professional field**			
	Practitioner	21 (47)	
	Researcher	19 (42)	
	Other	5 (11)	

### Principal Findings

#### Themes

Our principal findings are organized into the following 3 sections: (1) ways in which mobility data were used in response planning and policy making, (2) how data were processed, and (3) human factors that contributed to successful use (Table 2). The findings presented are resultant of triangulation from interviews from different groups (ie, researchers, practitioners) and reflexivity in the determination of themes.

**Table 2:** Taxonomy of themes by research question.

Research question	Themes
How groups use human mobility data and its results in response planning and policy making.	There was a perception that mobility data analyses could or did improve situational awareness. There was uncertainty on how results were ultimately used.
How mobility data were processed and ways of working	Misaligned data purpose with priorities was common. The team had difficulty interpreting meaning from results. Some teams determined the results were of limited value.
Human factors enabling or hindering the use of data	Human factors play a significant role in how mobility data were used. Researcher-practitioner teams that were more successful had open communication styles, enabling near real-time learning. Adapting processes, which was dependent upon communication, was often required to use the data successfully.

#### Use in Response Planning and Policy Making

Researchers and policy makers in the CMDN used mobility data to improve understanding of population movement; influence resource provision; and influence policies, specifically stay-at-home orders. Themes emerged among these narratives related to (1) the perception that mobility data analyses could or did improve situational awareness, (2) uncertainty on how to interpret mobility data results, and (3) the perceived lack of value despite understanding the results.

Response planning and policy making are often related but distinctly different activities. Response planning involves tactical, action-oriented activities such as daily planning and logistics, while policy making focuses on planning and articulation of actions to guide involved stakeholders who are often required to abide by or execute these policies. This qualitative study revealed that, for both response planning and policy making, there were no common uses of these data across user groups. In general, those interviewed in the study sought to better understand where people were moving as well as understand situational changes in their specific locality (eg, national, county, and city levels).

Mobility data helped many teams better understand specific group movements and helped others allocate resources such as personal protective equipment (PPE) and plan food distribution sites. One team used the data to further understand the movement of essential workers with the intention of better understanding disease spread in a large city. The mobility data helped them rank neighborhoods that had larger percent changes of movement during the citywide stay-at-home order compared with movement patterns before the pandemic (ie, baseline).

The mobility data has been used, and especially to identify the neighborhood that has [a] high proportion of essential service workers who had to report to work during [a] surge of COVID-19 cases. [We] calculated percent change of the commuting patterns during Week 13–the morning commute time, which is almost the end of March and all of April, the exact time the[city] experienced [a] huge surge of COVID-19 cases.

Practitioner, United States

Other government offices described using mobility data for PPE distribution along with other data sources to help with situational awareness and planning.

The [County] Office of Emergency Management is building an early warning system of areas that could become hotspots with the potential to overwhelm the medical and public health systems. Density and mobility are key indicators in this customized early warning system. We infuse the mobility data from these reports into a larger system that helps anticipate areas of potential viral spread, thus allowing for a more proactive response in regards to PPE supply distribution, and targeted community testing.

Researcher, United States

Interviewees mostly described how they intended to use mobility data, rather than reporting policy-related actions taken as a result of the data analyses. They also described how they used the data to plan for reopening or relaxation of existing stay-at-home orders. Many other interviewees expressed uncertainty about how the data were actually used in policy making.

I share[d] it with our health director every week and with the rest of our data group. And I think the thing that they're just mostly looking [at] when we move to phase one of the reopening, like how much did people start moving around more or were people still staying at home. And I think from this, we concluded that people were starting to move more.

Practitioner, United States

So, one of the things we can do with it is show that if you allow unrestricted travel during the Eid holidays, this outbreak, it's gonna spread really fast and it's gonna spread everywhere.” And so, they got the CDR (call detail records) data. We did some modeling. We showed what would happen and I'm not gonna say that that's what influenced them, but they did extend the lockdown so that people couldn't travel during the Eid holidays.

Researcher (located in the United States), India

#### Improving Situational Awareness

Although the Use in Response Planning and Policy making section describes specific use cases, interviewees often described how the data were used for general situational awareness. Many believed that the data could help them understand movement of people during stay-at-home orders or during relaxation of these orders. Some groups felt the data improved their situational awareness of localized outbreaks of COVID-19 and future COVID-19 transmission monitoring.

Facebook has actually given us the ability to not just look at overall levels of movement, but to be able to look at where people are moving from and to over the space of time. Because we've had these fairly localized point outbreaks.

Researcher, Australia

We've been able to use that data to put together essentially risk maps saying, okay, given that we saw transmission there last week, let's go back and look up what the transmission patterns in and out of that area, wherever the proceeding couple of weeks.

Researcher, Australia

These are 2 examples where mobility data enabled understanding of the movement of people over space and time, as well as transform their situational awareness into risk maps, a form of visualization. Although many described the data influencing their situational awareness, there were no findings that revealed accounts of explicit changes in decision-making.

#### Uncertainty

This section presents the multifaceted nature of not only understanding how mobility data were used in this disaster but also the uncertainty that individuals faced that became barriers to data use. There was significant uncertainty around how to transition from the idea that mobility data could be useful to actually transforming the data to assist in response planning and policy making. For example, even though practitioners and policy makers believed that mobility data could improve situational awareness of when and where people were moving, there was uncertainty around how to formulate a specific way in which mobility results could influence response planning.

I mean, [public health scientist] has told us repeatedly, this is very helpful for them to know...Exactly how to then use it for their response, I think that is a much harder question.

Researcher, India

And, so, they came up with their recommendations… and unfortunately, I was not involved with the direct communication with the data requesters…so my answer to your question is, I don’t know how the result of the analysis has shaped any policy decisions and any change of the course of actions.

Practitioner, United States

So, I don’t know exactly how he’s been using it. I know he’s been monitoring it just to see whether there are any places that raise any flags.”

Practitioner, United States

These excerpts reflect how both researchers and practitioners felt they did not know or lacked confidence on the exact use of the data for response planning or policy making. It remains unclear whether this uncertainty is due to limited skills in problem formulation with novel data, unfamiliarity with analytical methods, or challenges with communicating results.

### Processes and Ways of Working

#### Themes

This section describes how mobility data were processed. We present 4 ways of processing the data that respondents described: (1) identifying data purpose, (2) tools and products, (3) data preparation, and (4) results and data sharing. The themes that emerged were (1) the initial purpose for using mobility data was often misaligned with groups’ priorities, (2) results sharing was often limited when the results lacked meaning, and (3) the results were deemed of little or no value.

#### Identifying Data Purpose

In order to use mobility data, understanding the purpose of use is one of the first crucial steps. The study revealed that each team’s intention to use mobility data was linked to their perceived purpose of using the data. Although seemingly intuitive, it remains an important distinction. Many researchers and practitioners aimed to use the data to better understand where people were moving to and from and if groups were abiding by policies such as movement restrictions or stay-at-home orders. Others sought to correlate mobility data with other data sources to better understand the dynamics of COVID-19 disease transmission.

Most researchers relied upon practitioners to identify the specific purpose of using mobility data in their regions or localities and awaited direction from them. Some teams were able to identify specific questions for the data but often adapted their questions over time. In many circumstances, this was due to changing environments and, more importantly, an evolving understanding of what the mobility data could and could not answer for their specific questions.

Initially we were interested in...are people staying home, but then as we started to phase open, we also started tracking it even more closely to see, okay, now that we’re opening, are we seeing [that] more people are moving about and things like that.

Practitioner, United States

I think the ask has changed a little bit around what we’re layering the data with. The initial hope was to work with the county health department to better understand cases and different rates that we could correlate the mobility with. So, we could say, people were extremely mobile here, and we’re seeing a spike in cases later on in these specific hospitals. Unfortunately, we weren’t able to get that second piece of data, and with the aggregated data that we get from the county about the city as a whole we aren’t seeing any correlations with that.

Practitioner, United States

Other teams did not ultimately share the results of the data because they were either unable to confidently interpret the results or felt that the results were not relevant to their needs and priorities.

We’re not really sure how to move forward with the public health department I think, because we’re still unclear how exactly we should use this data to prioritize any sort of EpiModel.

Researcher, United States

...But the reaction, I remember when I first saw this [visualization] was, “Cool visualization. But, what am I gonna do with this? And what is it actually going to tell me?

Practitioner, United States

These excerpts show the diversity of data purposes among groups. Some groups’ data purpose evolved over time due to environmental changes or their ability to use the data with other sources.

#### Tools and Products

There was no standardized way researchers and practitioners teams used data tools, analysis software, or visualization platforms for their collaborative efforts. Ultimately, tools and products were described as vehicles with which to share information with other data teams, policy makers, and response planners. Some groups used geographic information systems (eg, ArcGIS software) and online mapping tools (eg, OpenStreet map), while others used data visualization tools such as Tableau and ShinyApp. Some products were spreadsheets (eg, csv files) that were shared by researchers to data teams within government offices. In some instances, both researcher and practitioner interviewees felt that providing analyses in these data formats helped practitioners create final reports.

So, I do know one outcome of that is that we requested that we get the all-city data from each timeframe and [he] was able to also upload that as CSV for us too, and that lines up with the PDF. So, at one point, we were trying to replicate what the PDFs had and couldn’t, and that actually helped us lead to asking for the data.

Practitioner, United States

#### Data Preparation

The components of data preparation include data cleaning, validation, and transformation, often bringing together different data types from various sources [33]. Mobility data were a novel data type for most researchers and practitioners and required them to take additional time to familiarize themselves during the data preparation process. Some interviewees described the preparation stage of “cleaning” mobility data, such as changing formats and structures to more easily align with other data sources to make the analysis relevant for their purposes.

In fact, we also asked them to change a little bit in terms of the columns and the rows that we wanted them to change because they were not compatible as Interviewee 067 was mentioning. So, we have to do a lot of work by ground.

Researcher, India

Many groups prioritized preparing the mobility data to better meet their local environment. They often reworked the data to meet their planning geography, whether it was formal administrative units or planning geographies such as jurisdictions*.*

There’s one for [city location]. There’s one for [neighborhood]. All of these different areas are more canonical for people. So, the data we receive are mapped to these, and it makes a lot of sense to people used to working with this geography. So, so long as all the other sources can be mapped to the same thing, that would probably make sense to the city and decision makers.

Volunteer, United States

For many groups, tailoring and preparation meant just learning more about what the data represented and becoming more familiar with mobility data. The process of preparing the data was in and of itself a way to learn and gain knowledge about the data and what it could ultimately mean for policy making and response planning.

Yeah, so we spent about five, six days working on the data and trying to understand what it represented. So, it’s probably a lot of back and forth with various people and from Facebook.

Researcher, Thailand

These excerpts reflect the need for teams from different contexts to further adapt the data to meet their specific needs, which took time to prepare.

#### Results and Data Sharing

Results were shared with national ministers, district mayors, emergency response teams, public health teams, and the European Commission, to name a few. Researchers frequently shared analytical results with practitioners in multiple formats (eg, graphs, maps) and sometimes even spreadsheets. Simplification, context alignment, and narrative text were often necessary to help readers understand what the results meant. For example, a data fellow in a city office simplified graphs to help her colleagues better interpret the results, anticipating areas of confusion and working to mitigate them. Other teams created their own charts from the data analysis to better visualize and share the results.

And if you can imagine a figure with a lot of noise, individuals that maybe don't have the experience that I do or people in the network have, may find that confusing. So, I did very simple things. I apply some smoothing algorithm so as to show trends rather than actual aggregated data. And that way I could mitigate the confusion regarding noise from the data...

Researcher, United States

First we look at the report that you [guys] send us. We take a look at that, understanding a little bit better. And then whatever insights that we gather from that report, I translate those into our actual report that we hand out to the city manager and use the CSV to create those charts.

Practitioner, United States

These excerpts reflect individuals and teams tailoring results or translating analytical findings to address “noise” or “confusion” with the aim to improve understanding and sharing with others.

Narrative statements accompanying analytical results were also common as practitioners sent reports to response planners and policy makers. Many felt that adding summaries or explanations helped transform the data results into meaningful information. Others used bullet formatting to help prioritize findings but included more lengthy results to be reviewed at the discretion of the end user.

And if we were sharing this report more broadly with our policy folks, that’s what they’re looking for, is that summary. But I think I shared our 20-some odd page weekly, right? So, we do that same thing. Here’s the top five or six bullets you need to know about and validate the rest if you want.

Practitioner, United States

Teams needed not only to communicate and share results with one another but also the skills and time to interpret the results and understand their meaning. Some challenges laid in understanding the mobility metrics, such as how movement was measured in relationship to trips taken during a time period.

So, these [are] some insights...that were not provided by the original graphs. And then, another thing that I thought was very insightful is this...when you stratify by districts, you see that it's driven mainly by these three districts up here. Some districts that are behaving, if we can say that, are adhering to the social distance policy.

Researcher, United States

Some lacked confidence in sharing the results with response planners and policy makers. Others described uncertainty in their interpretation of results and felt that the risks of sharing this information were too great to take on. Ultimately, this prevented many interviewees from sharing results with decision makers.

I guess the thing about this data is that it is really difficult to know how to interpret any one city, or any one weekend change. And I would be cautious about advising changes on that until we have more data on what that change actually represents.

Researcher, United States

Other environmental factors such as information and data overload prevented many from not only understanding the results but also identifying their value for decision-making. For many, understanding the data and translating the findings for others required significant time, often time that many did not feel they had.

This is a pretty detailed report, and I think some people looking at it would be kind of intimidated by it or wouldn’t have the time to really be able to go in and dig in deep, so I think we have thought a lot about how to give high-level updates that are meaningful and impactful.

Practitioner, United States

These excerpts highlight that additional processing was needed to distill results in order to feel comfortable sharing them. Different groups created different products to best meet the needs of their specific users and decision makers.

### Human Factors

#### Themes

Interviewees spoke about human factors, including data translation, communication, and adaptation, that contributed to successful use of mobility data. The following 3 themes emerged: (1) human factors played a significant role in how mobility data were used, (2) researcher-practitioner teams that were more successful had open communication styles, enabling near real-time learning, and (3) adapting processes was often required to use the data successfully, which was dependent upon communication.

#### Data Translation

Data translation skills include behaviors such as communication skills and the ability to communicate both technical aspects of data (eg, analysis, metrics) and relevant information meaning and value of the data itself in various formats. Data translation in the context of using mobility data during the COVID-19 pandemics was tightly linked to human information processing abilities and creating products that could provide situational awareness for response planners and decision makers.

And so, I think for us, that was the key thing: that the [researcher] was willing to do, and work with us on, and understand that…it was a process that we all had to work through, and he, [was] just available and willing to work with us and willing to help us navigate this new type of data.

Practitioner, United States

Data translation helped both researchers and practitioners understand the specific purpose for which to use the data. This enabled teams to refine geographic approaches to meet city, county, and regional contexts during stay-at-home orders and the many evolving COVID-19 epidemiologic environments, including relaxation of restrictions. Although team communications were primarily remote, these social interactions were viewed as important in enabling teams to understand what data opportunities lay ahead and adapt to changing environments, evolving collective knowledge about the data’s value, and optimal methods to present results to decision makers.

#### Communication

Teams communicated using different modalities and frequencies. Some groups had daily calls early in the collaboration, others connected on weekly Zoom meetings, and some primarily communicated via email. Most researchers did not communicate directly with response planners and policy makers but rather with data teams in government offices. The few that shared results directly did so via situation reports, updates in daily emergency operational cell meetings, or via WhatsApp and email. In South America, communications were via WhatsApp and direct voice communications from the researcher to the scientist in the country and then to national ministers.

I am part of [a] directory of people that are indirectly [in] discussion with the minister of science...I sent him an email directly to the minister and he ignored me. The same Professor said, well, it's easier if you [to] send me the reports and I am [in] a channel of communication with him.

Researcher, South America

Trusted relationships played a role in successful communications and often required some adaptations as teams determined more effective ways of sharing the results.

Although the route of communication was important, interviewees described the nature of communication including listening and back-and-forth communication as essential parts of a successful collaboration.

I don’t know, it’s so boring and simple, but I think it’s just about listening, and just keep asking questions to really understand where they’re at. And I think I was really helped along too by the communications director, because she was really open about asking questions when she didn’t understand things.

Researcher, United States

Communication often led to changes in ways of working and adapting approaches, such as redefining the purpose for using mobility data, simplifying results, and helping better translate the analytical results into meaningful findings that could be shared in a more understandable way.

#### Adaptation

Researchers and practitioners adapted their approaches, workflows, and communications to align with the local context. Over the course of the pandemic, response activities and policies in many jurisdictions changed, requiring many groups to reassess their original purpose for using mobility data. One team adapted their initial purpose from monitoring movement to monitoring movement after various restrictions were being lifted. Some increased the frequency of reporting as one jurisdiction went into lockdown. Others adjusted their approach to analysis once they gained a deeper understanding of what the data could and could not be used for.

I think the teams that I’ve had the best experiences with in terms of the use of these data and the teams that have also gotten the most out of it have been the ones where there have been these sort of clear lines of communication, and ability to change and upgrade things as we move forward.

Researcher, multiple locations

I’ve been asked to provide a second report. So, I shared the presentation I gave this morning, but I also wrote a report similar to this, since they’ve just gone into lockdown...So, usually, they’ll ask for a report on a monthly basis, – but when something happens, I try to be ready to give those as they request and have it ready to give it as quickly as I can.

Researcher, Botswana

These excerpts are evidence of adaptation being facilitated by strong and open communication.

## Discussion

### Principal Findings

This qualitative study, grounded in a phenomenology approach, analyzes the perceptions of researchers and practitioners who used mobility data during the first year of the COVID-19 pandemic. This study contributes to the emerging knowledge on how groups in disaster settings are using novel data sources for decision-making, specifically response planning and policy making. It also highlights the importance of human factors (ie, communications, data translation, and adaptation) as critical for the success of individuals and groups in defining a purpose for which to use novel data, identifying value in the data results, and ultimately using data in disaster response activities.

Although some groups used mobility data for distributing resources, most were unsure of how these data directly affected policy making. Evidence-based decision-making for policy making during COVID-19 remains an area of investigation, and published studies support our findings of limited use. A qualitative study of the perceptions of scientific experts and advisors in 11 countries described how evidence was thought to play a small role in overall policy making [34].

Humanitarian studies of data use in disaster environments have shown that, often, those who are managing, analyzing, and sharing data are often not the same individuals who are making decisions in emergency operations centers or leadership meetings [35]. The individuals we spoke to indicated this was the case in their partnerships as well. With novel data, it can take time to understand what the data represent and how the results are best communicated, requiring data translation skills or roles to help make the collaborations successful. Over time, the researchers and practitioners who were able to communicate and learn from one another were able to make the results more digestible for decision makers, likely due to improved knowledge and understanding and confidence in communicating this information.

There was a common understanding that groups were seeking new data sources to help improve situational awareness. This is in alignment with published literature on data-seeking behaviors and situational awareness. Endsley [36] described the theory of situational awareness in which individuals are described as not just recipients of data but active seekers who align this behavior with their current goals. However, with novel data, bidirectional communication between researchers and practitioners helped build collective knowledge of what the data could actually be used for in their local context. In addition, the direct collaboration between researchers and practitioners enabled teams to adapt quickly along various lines to try to meet the needs of each government group. Morss et al [37] noted that generating usable information among diverse decision makers requires working directly with these users to understand needs and context.

Human factors such as communications as well as adaptive approaches to data analyses and visual presentation were mentioned by many interviewees as steps along the way that helped them better prepare the data results for their intended users. These “soft skills” (ie, communication, interaction, creativity) described by Polese et al [38] are needed in conjunction with “hard skills” (ie, technical, analytical) to use big data. Soft skills are critical factors for the effectiveness of data interpretation and extraction of knowledge [38].

Our study identified a key role among teams—a data translator. Studies on big data note the importance of interdisciplinary and multistakeholder interaction and collaboration to enable more useful interpretation of big data [38]. Humanitarian and disaster organizations have increasingly become aware of the importance of data translators in leveraging big data opportunities [39,40]. Technology companies engaged in social good and business enterprises also recognize the role of data translators to meet their mission and goals [41-43]. Particularly when engaging with a novel type of data, this crucial role requires significant technical knowledge as well as an understanding of how the data can be best used. Often, this requires a shift in mindset for data translators to communicate simple, actionable information and communicate confidence in results. Other published studies on knowledge transfer between researchers and practitioners highlight the importance of social interactions and the social process that occurs in the environment of data, disasters, and decision-making [29,44].

### Limitations

Though qualitative research using near real-time data from an unprecedented global disaster has unique value, it faces challenges and limitations in data collection, bias, and generalizability. This study was limited to the first year of the COVID-19 global pandemic and does not reflect evolutions of the policy making and response planning among those interviewed over the course of the pandemic. Although Facebook (Meta) data reflect digital movement of individuals during disasters, other mobility data were not included in this study. Facebook’s history of collaborating during disasters and building the Facebook Data for Good program with anonymized and privacy-preserving data sources provided a valuable data set for our exploratory study.

Response bias may also have played a role with the interviewees, but all attempts were made to interview individuals and groups during the period of analyses to ensure information was gathered as proximate to the experience as possible. All interviews were conducted in English, which may bias the results toward the perceptions of individuals and groups who are proficient in this language. Although much research in data and disaster focuses on the impact on decision-making, this study intentionally did not explore direct decision-making. Rather, it focused on exploring and further understanding the preceding processes and human factors that could potentially facilitate or hinder the use of data. It also explored precursors of decision-making, which revealed a notable degree of uncertainty among this interview cohort on actual decision-making.

### Conclusion

This phenomenological study using a constructivist perspective with data from the early COVID-19 pandemic environment contributes to the understanding of how novel data sources are used in disaster settings. This study offered a unique opportunity to investigate the application of a novel data source during an unprecedented emergency. This resulted in the challenges of working with, understanding, and communicating these data while simultaneously figuring out how to apply the data for response planning and policy making. The groups we spoke to shared a broad range of perceptions and experiences related to these data, something that is expected in an emergency or disaster setting as well as with a novel data set.

This exploratory study highlights human factors as important facilitators for understanding data purpose, eliciting meaning from the data, and using the data for response planning and policy making. Although there were a few examples of how mobility data were used among specific researcher-practitioner groups, most groups were only able to describe their journey in exploring and learning about the data and share some of the barriers to use. Ultimately, the role of a data translator was crucial for understanding the complexities of this novel data source; adapting workflows, visualizations, and reports to align with end users and decision makers; and communicating this information meaningfully to address the goals of responders and policy makers.

This study complements the larger body of literature evaluating the use of big data, including mobility data during disasters, and highlights the need for a more qualitative perspective on use and value rather than quantitative metrics. Future studies that assess the use of data during disasters should include a focus on human factors, to further understand the role of communication and learning frameworks across diverse collaborative groups, specifically to understand the working relationship between members of these teams and how they navigate roles, expertise, and power in order to achieve their goals. Finally, further qualitative studies are needed to understand more fully the transition from perceived usefulness to decision-making to action in disaster settings.

## Appendix

Multimedia Appendix 1 Interview guide.

## Abbreviations

CMDN: COVID-19 Mobility Data Network
PPE: personal protective equipment
