# Supplementary material for: Understanding the Use of Mobility Data in Disasters: Exploratory Qualitative Study of COVID-19 User Feedback
Source: JMIR Hum Factors. 2024 Aug 1;11:e52257. doi: 10.2196/52257 (PMC11327621; doi:10.2196/52257)
Supplement: Multimedia Appendix 1 [file humanfactors_v11i1e52257_app1.docx]

Interview Questions

**General Questions**

**Overview and Scope of Activity**

- Can you tell me how your relationship with the COVID 19 Mobility Data Network began?
- What is your background? (Role, data involvement, disaster experience)
- Who do you work with from the network and what roles do you all play? Have they changed?

**Researcher Questions**

# In your opinion what do you think your collaborators in the county/state office expected to gain from mobility FB data?

# Who are you sharing situation reports with? Other products?

# How are people using the situation reports (Sitreps)?

# How did you choose your geographic units/metrics and approach to visualizations?

# In your opinion what has been most useful for those who you are trying to support?

# What have been the challenges in meeting the needs of those who you support?

# What are the requirements with respect to keep this sustained with your collaborations going forward?

- What would you say/what advice would you give to someone who is starting a similar project. What are the wins/challenges?

## Practitioner Questions

- Initially, how did you think the Facebook data could help your [X] during the COVID-19 response?
  - How did your understanding of the facebook data use change over time? (Calls, emails, meetings, etc)

# How often and through what route do you receive situation reports (Sitreps)? Other products?

# Please describe how you use the situation reports (Sitreps)?

- How do you think your colleagues are using the sitreps?
- The data/sitreps are presented in X geo unit (county, neighborhood, etc). Is this a good fit for your needs and those you work with for decision-making?
- In your opinion what (product) has been most useful for those who you are trying to support?
- What have been the challenges in meeting the needs of those who you support?

#####

What would you say/what advice would you give to someone who is starting a similar project. What are the wins/challenges?
